# The Cultural Evolution of Democracy: Saltational Changes in A Political Regime Landscape

**DOI:** 10.1371/journal.pone.0028270

**Published:** 2011-11-30

**Authors:** Patrik Lindenfors, Fredrik Jansson, Mikael Sandberg

**Affiliations:** 1 Centre for the Study of Cultural Evolution, Stockholm University, Stockholm, Sweden; 2 Department of Zoology, Stockholm University, Stockholm, Sweden; 3 Division of Applied Mathematics; School of Education, Culture and Communication, Mälardalen University, Västerås, Sweden; 4 School of Social and Health Sciences, Halmstad University, Halmstad, Sweden; University of Maribor, Slovenia

## Abstract

Transitions to democracy are most often considered the outcome of historical modernization processes. Socio-economic changes, such as increases in per capita GNP, education levels, urbanization and communication, have traditionally been found to be correlates or ‘requisites’ of democratic reform. However, transition times and the number of reform steps have not been studied comprehensively. Here we show that historically, transitions to democracy have mainly occurred through rapid leaps rather than slow and incremental transition steps, with a median time from autocracy to democracy of 2.4 years, and overnight in the reverse direction. Our results show that autocracy and democracy have acted as peaks in an evolutionary landscape of possible modes of institutional arrangements. Only scarcely have there been slow incremental transitions. We discuss our results in relation to the application of phylogenetic comparative methods in cultural evolution and point out that the evolving unit in this system is the institutional arrangement, not the individual country which is instead better regarded as the ‘host’ for the political system.

## Introduction

Our aim in this study was to investigate the cultural evolution of democracy; how countries change from being an autocracy to being a democracy and vice versa. An important difference between biological and cultural evolution is that whereas biological evolution builds on existing structures and natural selection of randomly appearing novel variation, cultural traits can be designed and chosen, that is, selected through cognitive processes. Another important difference is that whereas biological inheritance is transmitted from parent to offspring (vertical transfer), cultural information can be transmitted between any human individuals and collectives of individuals (horizontal and vertical transfer), for example [1], [2], [3], [4] [5]. These differences open up for much more rapid changes and saltational leaps in cultural evolution. We test this by investigating all transitions to democracy ever recorded to see if these occurred through slow gradual reform or rapid changes.

In a similar study of political complexity in Austronesian societies, Currie et al. [6] mapped political system onto a well-supported language phylogeny [7], thus assuming that political complexity spreads vertically, through inheritance over time, while horizontal transmission, through societies learning from each other, plays a minor role. They concluded that political complexity has historically risen and fallen in a sequence of small steps, mainly within societies. For democratization, however, we have access to the exact historical sequences of institutional change, enabling us to investigate the question of amount of horizontal transmission directly.

Transitions to democracy, which is typically defined as an “institutional arrangement for arriving at political decisions in which individuals acquire the power to decide by means of a competitive struggle for the people's vote” [8], are commonly considered the outcome of historical modernization processes. Socio-economic changes, such as increases in per capita GNP, education levels, urbanization, and communication, have traditionally been specified to be correlates or ‘requisites’ of democratic reform [9], [10], [11], [12], [13].

As Dahl [14] has pointed out, and following him, Vanhanen [15], democracy can be generalized as an ongoing interplay between increased participation and contestation (politically as well as economically), and a variety of its institutional forms has evolved. Arriving at new and democratic institutional arrangements may require dramatic national upheavals from authoritarianism [16], [17], [18], followed normally by a period of habituation to – and consolidation of – the early democratic institutions [19]. However, several classic studies stress the importance of a ‘democratic culture’ for success in this endeavour [20], [21].

Some scholars object to an implicit assumption of a final victory of democracy in all regimes of intermediate and dubious political character [22]. On the other hand, considering the dramatic and wave-like diffusion of democracy in the world [23], [24], [25], [26], [27], [28], [29], [30], other scholars, by means of innovation diffusion models, have predicted a bright future for democracy on a world scale [31]. But looking closer into the important details in this diffusion, the number of years and steps required during transitions into democracy have never been studied comprehensively [32], which is the rationale for this study.

The diffusion of democracy in the world system of states has normally been analyzed as the cumulative change into one minimum value of democracy in the Polity data sets [25], [27], [28], [29], [30], [31]. Here, we test whether we find step-wise or incremental versus rapid or saltational transitions to and from democracy.

## Materials and Methods

We used the Polity IV Data Series [33]. This unique data set scores nations on political regime characteristics over the years 1800–2008. Scores are assigned to each nation in six variables, with three variables on executive recruitment: (1) regulation of chief executive recruitment, (2) competitiveness of executive recruitment, (3) openness of executive recruitment; one variable on independence of executive authority: (4) executive constraints (decision rules); and finally two variables on political competition and opposition: (5) regulation of participation, and (6) competitiveness of participation.

For each stage in time since 1800, all countries have been coded with these six component variables, making it possible to classify a political regime from 4,550 possible institutional arrangements, which in turn are weighted according to a specified algorithm to produce autocracy and democracy scores. The sum of these two scores translates to a polity score on a 21-point scale representing degree of autocracy and institutional democracy. As an exception, a country can also be considered in an ‘interruption’, ‘interregnum’ or ‘transition period’, which cannot be translated to a polity score. Countries with a polity score of −6 or lower are classified as autocracies, while countries with a polity score of 6 or higher are classified as democracies, and the ones in between as ‘anocracies’. We have utilized the exact dates provided in the Polity data set throughout our analyses, rather than using year-by-year data.

To identify possible adaptive peaks in the political regime landscape, we took note of all historical transitions in polity scores for all nations over all years covered by the data set. These transitions where then graphed, enabling us to visually identify adaptive peaks in the governance landscape. We then identified every case where a nation had changed from being an autocracy (polity score ≤−6) to being a democracy (polity score ≥6), possibly passing several anocratic institutional arrangements, and vice versa. The number of days each such transition had taken was recorded, as well as the number of reform steps each state had passed. This enabled us to calculate the median time and median number of steps that transitions to and from democracy have taken historically.

## Results

**Figure 1:**
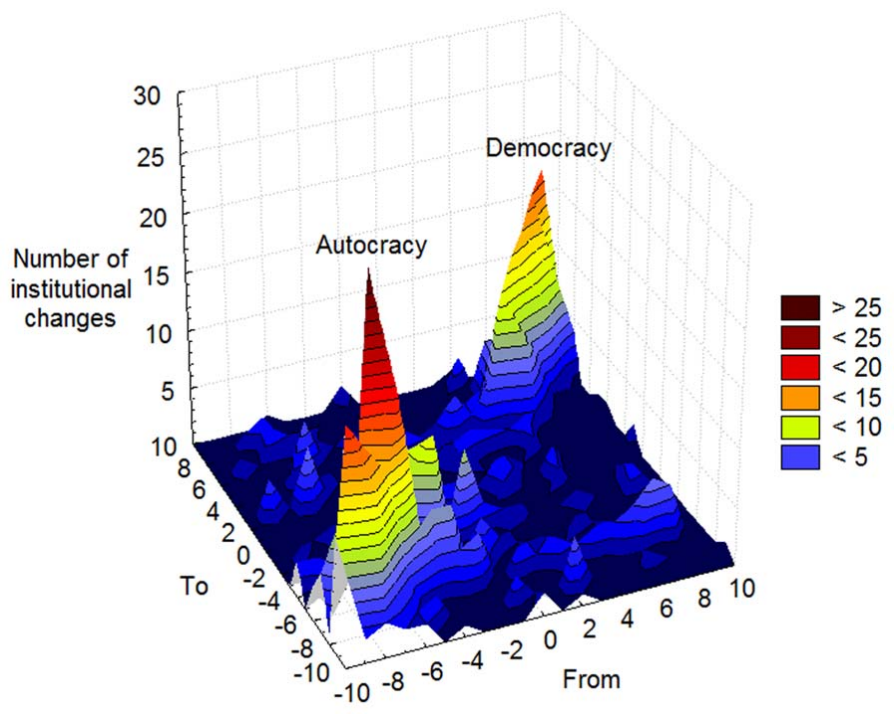
Two peaks in the evolutionary landscape of political regimes. We noted the source (‘From’) and target (‘To’) polity scores for all historical transitions in polity scores of all nations over all years present in the Polity IV data set. Since we only registered changes, the diagonal (i.e. 10→10, 9→9, 8→8, etc.) contains empty cells that are smoothed over in the graph. As the graph illustrates, changes in regimes tend to occur *within* autocratic or democratic types of regimes, and only seldom *between* them, or elsewhere in the space of possible transitions.

There are 122 recorded transitions between autocracy and democracy in the data, of which 79 are from autocracy to democracy, and 43 in the other direction. The data revealed that the average length of an institutional period (that is, a period without changes to any of the six variables) in polity is 9.3 years, with the median time between changes being 4.6 years. In the 860 cases that institutional transitions have occurred, however, a significant majority was towards increased democracy (511 cases; binomial test: p<0.001). Over time, such positive institutional changes in individual countries have resulted in an increasing number of democracies across the globe. Our analyses further showed that institutional transitions cluster in two peaks representing only the two major regime-types: autocracy and democracy. Change is much more common within these peaks than between them (Figure 1).

**Figure 2:**
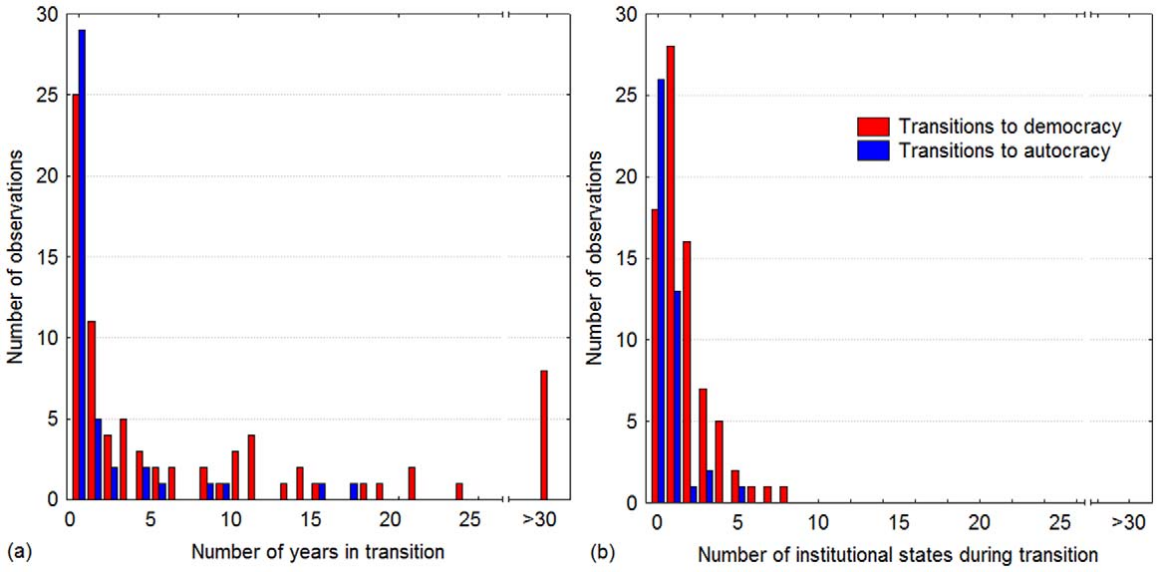
Rapid gains and losses of democracy. Changes between autocracy and democracy, and vice versa, tend to occur very quickly, both if (a) measured in units of time and if (b) measured in units of intermediate institutional arrangements.

When changes occur that move countries from autocracy to democracy, they tend to be very rapid. The median time required for moving a country from autocracy to democracy is 2.4 years, with 75% of these transitions occurring within 11 years (Fig. 2a). Similarly, the median number of intermediate governance states between autocracy and democracy is 1 state, with 78% of the changes occurring via at most 2 states (Fig. 2b).

Concerning the transitions in the other direction, from democracy to autocracy, the speed of change was even more pronounced. The median time required for moving a country from democracy to autocracy is 0 years, with 75% of the changes occurring within 1.4 years (Fig. 2a). A total of 93% of the changes occurred via at most 1 state (Fig. 2b). Although democratization is a rapid process, the relapse to autocracy is thus even faster.

As the previous results indicate, most transitions between democratic and autocratic types of political regimes occur through a single unique reform of the political system, or through a single intermediate state. Most of these changes via an intermediate step tend to go via a form of regime type defined in the Polity IV user's manual as a ‘transition period’. Such periods are described as ‘quite fluid, or volatile’, and ‘often result in unintended institutional arrangements’. Because of this definition, the ‘transition period’ is common en route from autocracy to democracy, and is an intermediate state in 53% of the transitions on the way to democracy and 26% of the transitions on the way to autocracy.

## Discussion

We found that autocracy and democracy have acted as peaks in an evolutionary landscape of possible modes of institutional arrangements. Movements between these peaks have occurred rapidly through direct reforms or via a short period of transition. Only scarcely have there been slow incremental transitions. Thus, democratization on a global scale the last two centuries has dominatingly occurred through rapid saltational transitions rather than through gradual incremental evolution.

Our results highlight two central differences between biological and cultural evolution. First, the governance system termed democracy – involving a large number of institutional rules and regulations – may be chosen by reformers and political actors. Minor institutional changes can then potentially be locally adapted to national conditions and therefore be modified and improved, but this is outside the scope of the Polity IV data set. In its major institutional foundations democratization has not been slow and incremental, but rapid and saltational, in waves of democratization [23], [24], [25], [26], [27], [28], [29], [30]. It is highly unlikely that reformers in several countries have invented democracy independently of developments in other countries, so democratization is likely to be a consequence of horizontal transmission. Indeed, other empirical studies support the conclusion that democracy diffuses horizontally (in particular, the study by Wejnert [28] is relevant here).

Second, the amount of horizontal transfer has been shown to be of importance for the reliability of results of phylogenetic methods in anthropology (e.g. [1], [2], [3], [4], [5]). In the case of democracy, horizontal transmission has been the rule rather than the exception, rendering phylogenetic methods at national level unusable. Actually, the birth and death of nations does not have a tree structure, as nations both merge and split. The potential of vertical transfer is highly limited. Indeed, since 1800, in only three cases have democratic countries split into two democratic countries (in 1993 when Czechoslovakia split into the Czech Republic and Slovakia, 2006 when Serbia-Montenegro split into Serbia and Montenegro, and in 2008 when Serbia split into Serbia and Kosovo).

A reconstruction of democracy as a political system on a language phylogeny would almost certainly indicate democracy as the ancestral state for large sections of the phylogeny. However, since we have exact information of all transitions, we know this not to be true. Instead all changes from autocracy to democracy have occurred within countries – reformers have learnt from or been inspired by the experiences of other countries.

This should serve as a sobering warning against applying phylogenetic models in studies of cultural evolution. For example, recently Currie et al. [6] mapped the evolution of political complexity onto a language phylogeny of the Austronesian languages. Implicit in applying phylogenetic methodology in that analysis, however, is the assumption that Autronesians have learnt very little from their neighbours. This may or may not be true, but it is an issue that should be investigated, not assumed.

In fact, there exist several clear examples where we know traits to have spread across ethnic borders in traditional societies, even though we do not have the exact sequence of events. When Europeans colonized America they brought with them horses. Native Americans were soon to acquire horses as well as the culture of herding and breeding these and quickly realized their utility for hunting, transportation and war. The use of horses rapidly spread across the many different cultures of Native Americans on the continent [34]. Travelling the other direction, maize from South America suited the climate and the needs of the African continent well. Today, maize is a staple in many African ethnic groups across the African continent [35]. Similar histories exist for potatoes, tomatoes, tobacco and cocoa.

These examples highlight that the analogy drawn from biological evolution, that ethnic groups/nations can be treated as evolving ‘species’, is questionable. A better analogy would be that the cultural traits are evolving units in themselves, with ‘morphology’ as given by their characteristics [36]. Ethnic groups/nation states are in this view better regarded as hosts, or ‘habitats’, invaded for shorter or longer periods by cultural traits. Once a cultural trait has evolved, it can spread rapidly across nations as part of a package of several interrelated cultural traits.
